# Supplementary material for: Over-Inflating a Tracheostomy Tube Cuff for Tracheo-Innominate Artery Fistula
Source: Diagnostics (Basel). 2024 Jan 20;14(2):223. doi: 10.3390/diagnostics14020223 (PMC10814070; doi:10.3390/diagnostics14020223)
Supplement: Supplementary file 1 [file diagnostics-14-00223-s001.zip › diagnostics-2806710-supplementary.pdf]

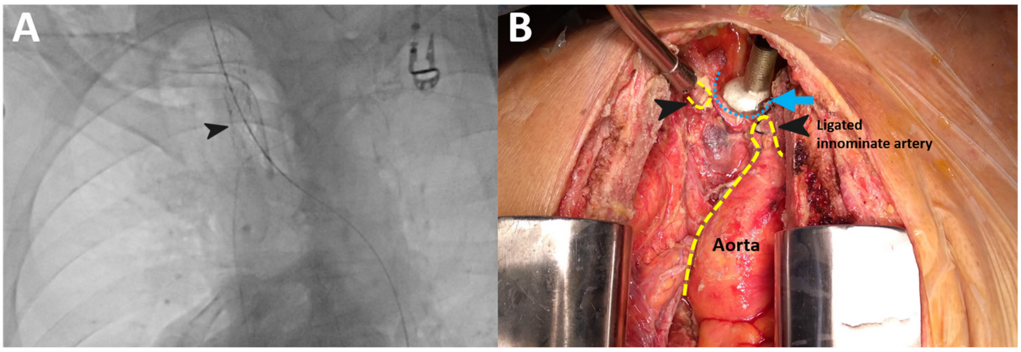

Figure S1. Angiography and intraoperative picture of surgical procedure. (**A**) Endovascular stent (arrowhead). (**B**) Ligated right innominate artery stumps (black dashed lines and arrowheads); a tracheostomy tube cuff (blue arrow) causes erosion of the trachea (blue dotted line).
